# 2-Hydrazinyl­quinoline

**DOI:** 10.1107/S1600536812026906

**Published:** 2012-06-20

**Authors:** Muhd. Hidayat bin Najib, Ai Ling Tan, David J. Young, Seik Weng Ng, Edward R. T. Tiekink

**Affiliations:** a Faculty of Science, Universiti Brunei Darussalam, Jalan Tungku Link BE 1410, Negara Brunei Darussalam; b Department of Chemistry, University of Malaya, 50603 Kuala Lumpur, Malaysia; c Chemistry Department and Faculty of Science, King Abdulaziz University, PO Box 80203 Jeddah, Saudi Arabia

## Abstract

In the title compound, C_9_H_9_N_3_, the 12 non-H atoms are essentially planar (r.m.s. deviation = 0.068 Å). The maximum deviation from planarity is reflected in the torsion angle between the β-N atom of the hydrazinyl residue and the quinolinyl N atom [N—N—C—N = −12.7 (3)°]; these atoms are *syn*. In the crystal, supra­molecular layers in the *bc* plane are formed *via* N—H⋯N hydrogen bonds.

## Related literature

For applications of coordination complexes of hydrazones as organic light emitting diodes and supra­molecular magnetic clusters, see: Zhang *et al.* (2011 ▶); Petukhov *et al.* (2009 ▶). For background to the synthesis of hydrazones, see: Gupta *et al.* (2007 ▶); Anwar *et al.* (2011 ▶). For a related structure, see: Najib *et al.* (2012 ▶).
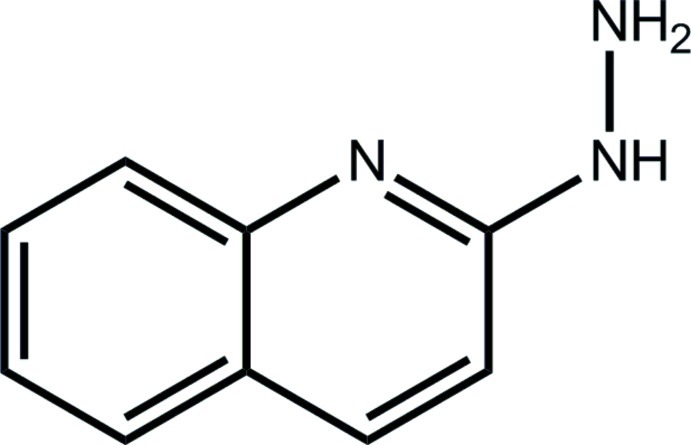


## Experimental

### 

#### Crystal data

C_9_H_9_N_3_
*M* *_r_* = 159.19
Monoclinic, 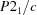

*a* = 13.7966 (9) Å
*b* = 3.9648 (3) Å
*c* = 14.0700 (8) Å
β = 97.039 (5)°
*V* = 763.84 (9) Å^3^
*Z* = 4
Cu *K*α radiation
μ = 0.70 mm^−1^
*T* = 100 K
0.30 × 0.08 × 0.03 mm

#### Data collection

Agilent SuperNova Dual diffractometer with an Atlas detector
Absorption correction: multi-scan (*CrysAlis PRO*; Agilent, 2012 ▶) *T*_min_ = 0.476, *T*_max_ = 1.000
2474 measured reflections
1542 independent reflections
1169 reflections with *I* > 2σ(*I*)
*R*_int_ = 0.018

#### Refinement

*R*[*F* ^2^ > 2σ(*F* ^2^)] = 0.052
*wR*(*F* ^2^) = 0.158
*S* = 1.10
1542 reflections
121 parameters
H atoms treated by a mixture of independent and constrained refinement
Δρ_max_ = 0.32 e Å^−3^
Δρ_min_ = −0.23 e Å^−3^

### 

Data collection: *CrysAlis PRO* (Agilent, 2012 ▶); cell refinement: *CrysAlis PRO*; data reduction: *CrysAlis PRO*; program(s) used to solve structure: *SHELXS97* (Sheldrick, 2008 ▶); program(s) used to refine structure: *SHELXL97* (Sheldrick, 2008 ▶); molecular graphics: *ORTEP-3* (Farrugia, 1997 ▶) and *DIAMOND* (Brandenburg, 2006 ▶); software used to prepare material for publication: *publCIF* (Westrip, 2010 ▶).

## Supplementary Material

Crystal structure: contains datablock(s) global, I. DOI: 10.1107/S1600536812026906/su2456sup1.cif

Structure factors: contains datablock(s) I. DOI: 10.1107/S1600536812026906/su2456Isup2.hkl

Supplementary material file. DOI: 10.1107/S1600536812026906/su2456Isup3.cml

Additional supplementary materials:  crystallographic information; 3D view; checkCIF report

## Figures and Tables

**Table 1:** Hydrogen-bond geometry (Å, °)

*D*—H⋯*A*	*D*—H	H⋯*A*	*D*⋯*A*	*D*—H⋯*A*
N2—H1*n*⋯N3^i^	0.93 (3)	2.18 (3)	3.077 (2)	164 (2)
N3—H2*n*⋯N1^ii^	0.89 (2)	2.31 (2)	3.200 (2)	175.1 (19)
N3—H3*n*⋯N2^iii^	0.90 (2)	2.58 (2)	3.295 (2)	136.4 (16)

Symmetry codes: (i) 
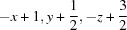
; (ii) 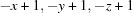; (iii) 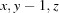.

**Fig. 1.**
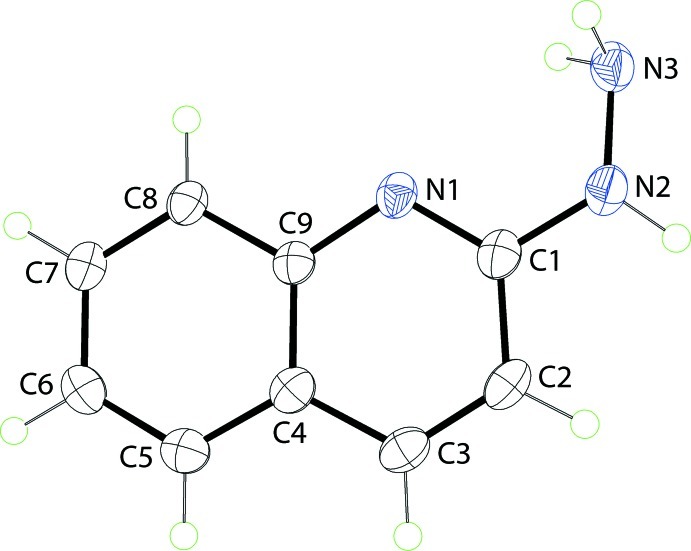
The molecular structure of the title molecule showing the atom-labelling scheme. The displacement ellipsoids are drawn at the 50% probability level.

**Fig. 2.**
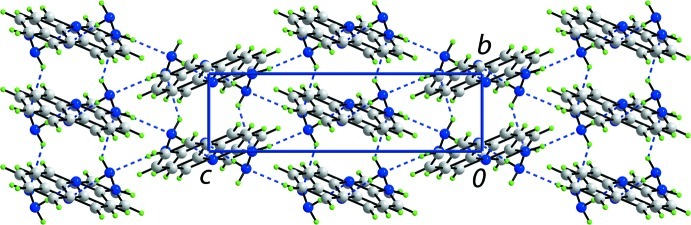
A view of the supramolecular layer in the bc plane in the crystal of the title compound. The N—H···N hydrogen bonds are shown as blue dashed lines (see Table 1 for details).

**Fig. 3.**
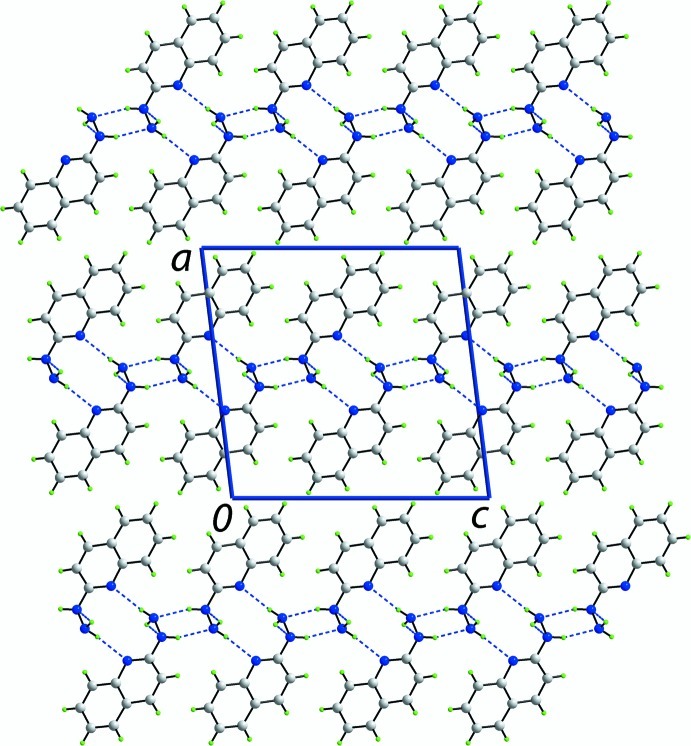
A view of the unit-cell contents of the title compound in projection down the b axis. The N—H···N hydrogen bonds are shown as blue dashed lines (see Table 1 for details).

## supplementary crystallographic information

### Comment

Hydrazones are versatile nitrogen donor ligands which have been used extensively for making coordination complexes for a variety of applications from organic light emitting diode (OLED) materials (Zhang *et al.*, 2011) to supramolecular magnetic clusters (Petukhov *et al.*, 2009). These ligands are made by condensation of a carbonyl compound with an organic hydrazine or hydrazide (Anwar *et al.*, 2011). We have previously reported the solid-state structure of the zinc(II) complex of 3,5-dimethyl-1- (2'-quinolyl)pyrazole (Najib *et al.*, 2012). The ligand in that complex was made by the condensation of acetylacetone with the title compound (Gupta *et al.*, 2007). Herein, the crystal and molecular structure of the title compound is described.

In the title compound, Fig. 1, the 12 non-hydrogen atoms are planar with a r.m.s. deviation = 0.068 Å and maximum deviations of 0.068 (2) and -0.152 (2) Å for the N1 and N3 atoms, respectively. The amine-N3 group is *syn* with the quinolinyl-N1 atom with the N3—N2—C1—N1 torsion angle being -12.7 (3)°.

In the crystal, molecules assemble into supramolecular layers in the *bc* plane *via* N—H···N hydrogen bonds, Fig. 2 and Table 1. The secondary amine-H hydrogen bonds to the primary amine-N2 atom. One of the primary amine-H atoms forms a hydrogen bond with the quinolinyl-N atom and the other forms a weak interaction with the secondary amine-N2 atom. The layers stack along the *a* axis with no specific interactions between them, Fig. 3.

### Experimental

The title compound was prepared by modification of a literature procedure (Gupta *et al.*, 2007). 2-Chloroquinoline (10.06 g) and hydrazine monohydrate (64–65% N_2_H_4_) in water (10 ml) were refluxed for 2 h. The water was removed using a rotary evaporator to provide a scarlet residue which was triturated with water and filtered. This scarlet solid was recrystallized from CH_2_Cl_2_ and hexane to provide 6.48 g (66.6%) of the title compound [M.p. = 417 K]. Spectroscopic data for the title compound are given in the archived CIF.

### Refinement

C-bound H-atoms were placed in calculated positions [C—H = 0.95 Å, *U*_iso_(H) = 1.2*U*_eq_(C)] and were included in the refinement in the riding model approximation. The N-bound H-atoms were located in a difference Fourier map and refined freely.

### Crystal data

**Table:**

C_9_H_9_N_3_	*F*(000) = 336
*M**_r_* = 159.19	*D*_x_ = 1.384 Mg m^−^^3^
Monoclinic, *P*2_1_/*c*	Cu *K*α radiation, λ = 1.54184 Å
Hall symbol: -P 2ybc	Cell parameters from 799 reflections
*a* = 13.7966 (9) Å	θ = 3.2–75.8°
*b* = 3.9648 (3) Å	µ = 0.70 mm^−^^1^
*c* = 14.0700 (8) Å	*T* = 100 K
β = 97.039 (5)°	Plate, red
*V* = 763.84 (9) Å^3^	0.30 × 0.08 × 0.03 mm
*Z* = 4	

### Data collection

**Table:**

Agilent SuperNova Dual diffractometer with an Atlas detector	1542 independent reflections
Radiation source: SuperNova (Cu) X-ray Source	1169 reflections with *I* > 2σ(*I*)
Mirror monochromator	*R*_int_ = 0.018
Detector resolution: 10.4041 pixels mm^-1^	θ_max_ = 76.0°, θ_min_ = 3.2°
ω scan	*h* = −16→17
Absorption correction: multi-scan (*CrysAlis PRO*; Agilent, 2012)	*k* = −3→4
*T*_min_ = 0.476, *T*_max_ = 1.000	*l* = −17→17
2474 measured reflections	

### Refinement

**Table:**

Refinement on *F*^2^	Primary atom site location: structure-invariant direct methods
Least-squares matrix: full	Secondary atom site location: difference Fourier map
*R*[*F*^2^ > 2σ(*F*^2^)] = 0.052	Hydrogen site location: inferred from neighbouring sites
*wR*(*F*^2^) = 0.158	H atoms treated by a mixture of independent and constrained refinement
*S* = 1.10	*w* = 1/[σ^2^(*F*_o_^2^) + (0.1*P*)^2^] where *P* = (*F*_o_^2^ + 2*F*_c_^2^)/3
1542 reflections	(Δ/σ)_max_ < 0.001
121 parameters	Δρ_max_ = 0.32 e Å^−^^3^
0 restraints	Δρ_min_ = −0.23 e Å^−^^3^

### Special details

**Table:**

Experimental. Spectroscopic data for the title compound: IR \v/cm^-1^: 3282, 3188, 3042, 2954, 2926, 2854, 1621, 1529, 1462, 1404, 1377, 1307, 1146, 1116, 955, 816, 746. _^1^H NMR 400MHz (CDCl_3) δ: 7.82 (1H, d), 7.71 (1H, d), 7.60 (1H, d), 7.54 (1H, dd), 7.23 (1H, dd), 6.75 (1 H, d), 4.0 (3H, br s). ^13^C NMR 100MHz (CDCl_3_) δ: 158.8, 147.3, 137.4, 129.7, 127.5, 126.3, 124.2, 122.8, 110.6.
Geometry. All e.s.d.'s (except the e.s.d. in the dihedral angle between two l.s. planes) are estimated using the full covariance matrix. The cell e.s.d.'s are taken into account individually in the estimation of e.s.d.'s in distances, angles and torsion angles; correlations between e.s.d.'s in cell parameters are only used when they are defined by crystal symmetry. An approximate (isotropic) treatment of cell e.s.d.'s is used for estimating e.s.d.'s involving l.s. planes.
Refinement. Refinement of *F*^2^ against ALL reflections. The weighted *R*-factor *wR* and goodness of fit *S* are based on *F*^2^, conventional *R*-factors *R* are based on *F*, with *F* set to zero for negative *F*^2^. The threshold expression of *F*^2^ > σ(*F*^2^) is used only for calculating *R*-factors(gt) *etc*. and is not relevant to the choice of reflections for refinement. *R*-factors based on *F*^2^ are statistically about twice as large as those based on *F*, and *R*- factors based on ALL data will be even larger.

### Fractional atomic coordinates and isotropic or equivalent isotropic displacement parameters (Å^2^)

**Table:**

	*x*	*y*	*z*	*U*_iso_*/*U*_eq_	
N1	0.35068 (10)	0.3907 (4)	0.51371 (10)	0.0241 (4)	
N2	0.44545 (11)	0.4959 (4)	0.65825 (10)	0.0315 (4)	
N3	0.52127 (11)	0.2769 (4)	0.63649 (11)	0.0305 (4)	
C1	0.35855 (13)	0.5229 (5)	0.60074 (12)	0.0261 (4)	
C2	0.28104 (14)	0.6982 (5)	0.63892 (12)	0.0285 (4)	
H2	0.2907	0.7905	0.7017	0.034*	
C3	0.19420 (13)	0.7297 (4)	0.58430 (13)	0.0277 (4)	
H3	0.1422	0.8465	0.6084	0.033*	
C4	0.18015 (13)	0.5882 (5)	0.49047 (12)	0.0250 (4)	
C5	0.09162 (13)	0.6087 (5)	0.42974 (13)	0.0281 (4)	
H5	0.0375	0.7219	0.4510	0.034*	
C6	0.08226 (13)	0.4670 (5)	0.33990 (13)	0.0287 (4)	
H6	0.0220	0.4808	0.2995	0.034*	
C7	0.16246 (13)	0.3017 (5)	0.30833 (12)	0.0271 (4)	
H7	0.1558	0.2029	0.2464	0.033*	
C8	0.25074 (13)	0.2802 (4)	0.36567 (12)	0.0244 (4)	
H8	0.3044	0.1700	0.3428	0.029*	
C9	0.26140 (12)	0.4220 (4)	0.45841 (11)	0.0225 (4)	
H1n	0.4430 (19)	0.564 (7)	0.721 (2)	0.058 (7)*	
H2n	0.5538 (16)	0.370 (6)	0.5919 (16)	0.038 (6)*	
H3n	0.4950 (14)	0.090 (6)	0.6069 (14)	0.025 (5)*	

### Atomic displacement parameters (Å^2^)

**Table:**

	*U*^11^	*U*^22^	*U*^33^	*U*^12^	*U*^13^	*U*^23^
N1	0.0259 (7)	0.0276 (8)	0.0193 (7)	−0.0045 (6)	0.0042 (5)	0.0015 (5)
N2	0.0317 (8)	0.0407 (10)	0.0218 (7)	−0.0017 (7)	0.0016 (6)	−0.0034 (7)
N3	0.0277 (8)	0.0396 (10)	0.0238 (8)	−0.0043 (7)	0.0017 (6)	0.0025 (6)
C1	0.0283 (8)	0.0283 (10)	0.0222 (8)	−0.0074 (7)	0.0051 (6)	0.0016 (6)
C2	0.0377 (10)	0.0283 (9)	0.0208 (8)	−0.0052 (8)	0.0095 (7)	−0.0026 (7)
C3	0.0339 (9)	0.0252 (9)	0.0258 (9)	−0.0012 (7)	0.0111 (7)	0.0003 (7)
C4	0.0298 (9)	0.0228 (9)	0.0234 (8)	−0.0028 (7)	0.0069 (6)	0.0030 (6)
C5	0.0273 (9)	0.0266 (10)	0.0310 (9)	0.0005 (7)	0.0067 (7)	0.0039 (7)
C6	0.0253 (8)	0.0288 (10)	0.0312 (9)	−0.0017 (7)	−0.0005 (6)	0.0040 (7)
C7	0.0312 (9)	0.0286 (10)	0.0214 (8)	−0.0035 (7)	0.0026 (7)	0.0002 (6)
C8	0.0270 (8)	0.0266 (9)	0.0202 (8)	−0.0021 (7)	0.0046 (6)	0.0008 (6)
C9	0.0236 (8)	0.0232 (9)	0.0212 (8)	−0.0033 (7)	0.0051 (6)	0.0031 (6)

### Geometric parameters (Å, º)

**Table:**

N1—C1	1.324 (2)	C3—H3	0.9500
N1—C9	1.380 (2)	C4—C5	1.405 (2)
N2—C1	1.366 (2)	C4—C9	1.421 (2)
N2—N3	1.421 (2)	C5—C6	1.375 (3)
N2—H1n	0.93 (3)	C5—H5	0.9500
N3—H2n	0.89 (2)	C6—C7	1.404 (2)
N3—H3n	0.90 (2)	C6—H6	0.9500
C1—C2	1.434 (2)	C7—C8	1.379 (2)
C2—C3	1.348 (3)	C7—H7	0.9500
C2—H2	0.9500	C8—C9	1.412 (2)
C3—C4	1.426 (2)	C8—H8	0.9500
			
C1—N1—C9	116.89 (15)	C5—C4—C3	123.37 (16)
C1—N2—N3	122.42 (15)	C9—C4—C3	116.97 (16)
C1—N2—H1n	114.1 (16)	C6—C5—C4	120.82 (16)
N3—N2—H1n	119.8 (17)	C6—C5—H5	119.6
N2—N3—H2n	110.1 (15)	C4—C5—H5	119.6
N2—N3—H3n	109.6 (13)	C5—C6—C7	119.52 (16)
H2n—N3—H3n	102.9 (19)	C5—C6—H6	120.2
N1—C1—N2	118.89 (16)	C7—C6—H6	120.2
N1—C1—C2	123.91 (16)	C8—C7—C6	121.16 (16)
N2—C1—C2	117.19 (15)	C8—C7—H7	119.4
C3—C2—C1	118.89 (15)	C6—C7—H7	119.4
C3—C2—H2	120.6	C7—C8—C9	120.05 (16)
C1—C2—H2	120.6	C7—C8—H8	120.0
C2—C3—C4	120.13 (16)	C9—C8—H8	120.0
C2—C3—H3	119.9	N1—C9—C8	118.03 (15)
C4—C3—H3	119.9	N1—C9—C4	123.20 (15)
C5—C4—C9	119.66 (15)	C8—C9—C4	118.77 (15)
			
C9—N1—C1—N2	179.57 (15)	C4—C5—C6—C7	−0.4 (3)
C9—N1—C1—C2	−1.1 (3)	C5—C6—C7—C8	−0.3 (3)
N3—N2—C1—N1	−12.7 (3)	C6—C7—C8—C9	0.8 (3)
N3—N2—C1—C2	167.89 (16)	C1—N1—C9—C8	−179.37 (16)
N1—C1—C2—C3	0.7 (3)	C1—N1—C9—C4	0.4 (2)
N2—C1—C2—C3	−179.94 (16)	C7—C8—C9—N1	179.17 (16)
C1—C2—C3—C4	0.4 (3)	C7—C8—C9—C4	−0.6 (3)
C2—C3—C4—C5	179.51 (18)	C5—C4—C9—N1	−179.88 (16)
C2—C3—C4—C9	−1.0 (2)	C3—C4—C9—N1	0.6 (3)
C9—C4—C5—C6	0.6 (3)	C5—C4—C9—C8	−0.1 (3)
C3—C4—C5—C6	−179.92 (17)	C3—C4—C9—C8	−179.61 (15)

### Hydrogen-bond geometry (Å, º)

**Table:**

*D*—H···*A*	*D*—H	H···*A*	*D*···*A*	*D*—H···*A*
N2—H1*n*···N3^i^	0.93 (3)	2.18 (3)	3.077 (2)	164 (2)
N3—H2*n*···N1^ii^	0.89 (2)	2.31 (2)	3.200 (2)	175.1 (19)
N3—H3*n*···N2^iii^	0.90 (2)	2.58 (2)	3.295 (2)	136.4 (16)

Symmetry codes: (i) −*x*+1, *y*+1/2, −*z*+3/2; (ii) −*x*+1, −*y*+1, −*z*+1; (iii) *x*, *y*−1, *z*.
